# A case of *monocephalus* *rachipagus tribrachius tetrapus* in a puppy

**Published:** 2016-09-15

**Authors:** Adonis Pino, Arlenis Pérez, Aine Seavers, Guillermo Hermo

**Affiliations:** 1 *Private Veterinary Practitioner, Lanús, Argentina; *; 2 *Private Veterinary Practitioner, Oak Flats, Australia.*

**Keywords:** *Dipygus*, *Monocephalus*, Puppy, Twinning

## Abstract

Caudal duplication (*dipygus*) is an uncommon pathologic of conjoined twinning. The conjoined malformation is classified according to the nature and site of the union. We report the presence of this malformation in a female crossbreed puppy. The puppy was delivered by caesarean section following a prolonged period of dystocia. External findings showed a single head (*monocephalus*) and a normal cranium with no fissure in the medial line detected. The thorax displayed a caudal duplication arising from the lumbosacral region (*rachipagus*). The puppy had three upper limbs, a right and left, and a third limb in the dorsal region where the bifurcation began. The subsequent caudal duplication appeared symmetrical. Necropsy revealed internal abnormalities consisting of a complete duplication of the urogenital system and a duplication of the large intestines arising from a bifurcation of the caudal ileum***.*** Considering the morphophysiological description the malformation described would be classified as the first case in the dog of a *monocephalus rachipagus tribrachius tetrapus*.

## Introduction

Conjoined twining, is a structural congenital malformation. The exact mechanism by which caudal duplications originate has not been fully elucidated. Two theories, "fission and fusion", have been developed to explain the occurrence of conjoining.^1^^-^^3^ Fission theory proposes a failure in the formation of one or more constituents of the body during embryonic development,^4^ most likely if the embryonic disc divides later than the 13^th^ day post fertilization. Fusion theory proposes a fusion occurring between two originally separate monovular embryonic discs, therefore, embryos are blocked or subsequently joined by cell adhesion molecules in foetus.^2^^,^^3^

The conjoined malformation is classified according to the nature and site of the union e.g., craniopagus; joined at the head, thoracopagus; joined at chest, omphalopagus or xiphopagous; joined from sternum to waist, rachipagus; joined at the lumbosacral region.^5^ Although rare, the incidence of conjoined malformations in humans is estimated to be between 1/100000 to 1/50000 with the oldest recorded case dating back to 1100 A.D.^6^ Conjoined twinning occurs more frequently in cattle than other domestic species, but has been reported in birds,^7^^,^^8^ lamb,^2^^,^^9^ goat,^10^^-^^12^ cat^13^ and dogs.^14^^-^^16^

Dipygus (double buttocks), the only authentic variety of cephalic parapagia, refers to conjoined twins with the head and thorax completely merged with the pelvis and lower extremities duplicated.^11^^,^^14^^,^^17^^,^^18^

In this report we present an unusual form of dipygus twins joined at the lumbosacral region (rachipagus) with the presence of three upper limbs in a cross bred puppy.

## Case Description

In the present report, we describe a case of Mono-cephalus dipygus in a puppy. One week prior to presentation, echography on the bitch had identified four fetuses. The bitch commenced labor normally and initially delivered one live normal puppy in the first 2 hr of labor. When the labor failed to progress naturally, the bitch was referred to South Veterinary Clinic located in Lanus, Buenos Aires, Argentina. An examination revealed a puppy stuck in the birth canal and a caesarian was undertaken.

A malformed dead puppy removed from the birth canal was presumed to be the cause of the dystocia. Two other puppies were located, one on each horn of uterus. These puppies were extracted and given immediate assistance. Both were alive and appeared physically normal.

**Fig. 1:**
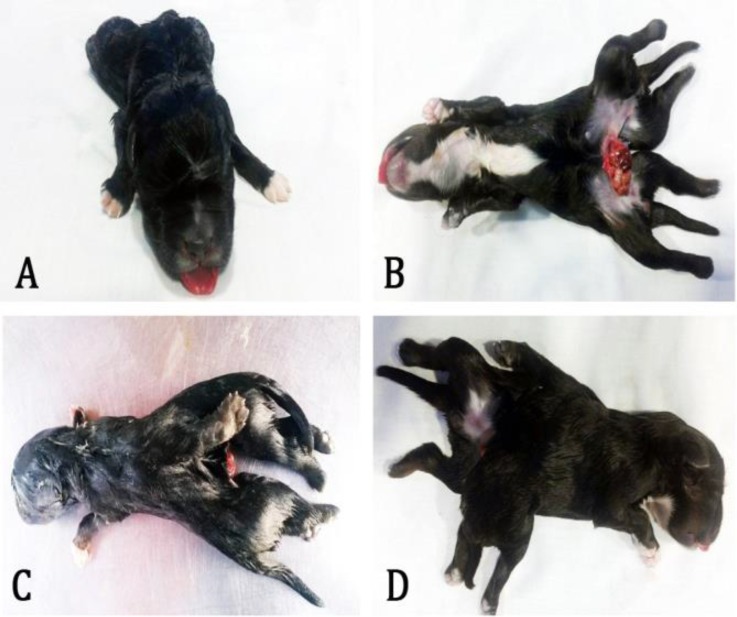
Different views showing the external features of puppy. (A) cranial view showing the absence of cleft palate, (B) ventral view showing complete caudal duplication, four lower limbs and two tails, (C) dorsal view showing supernumerary limb, (D) lateral view

**Fig. 2:**
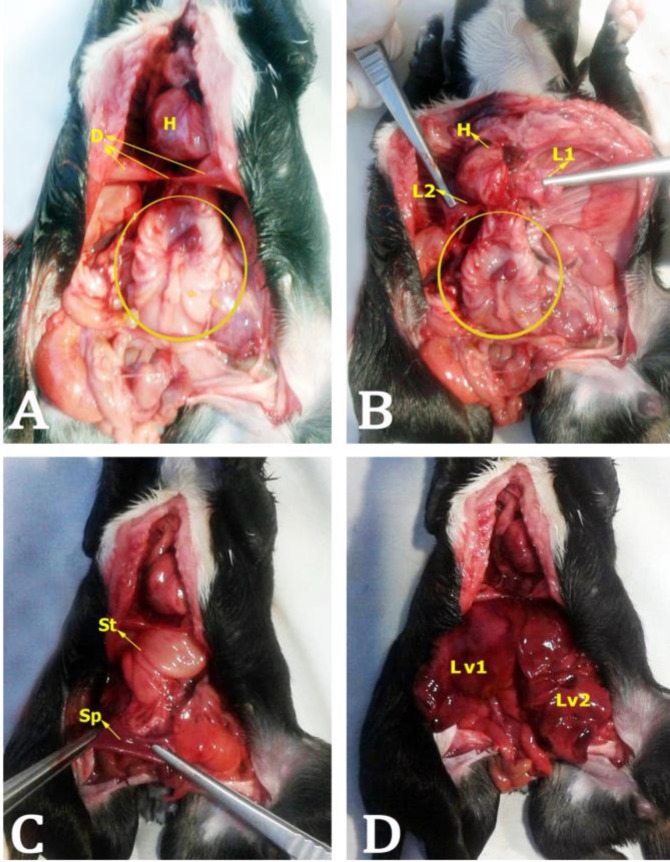
Union of the twins in the lumbosacral region (rachiphagus) and some internal organs. The site of join is marked by the yellow circle. A: Division of thoracic and abdominal cavity, (D) diaphragm, (H) heart; B: (L1) right lung, (L2) left lung; C: (St) stomach, (Sp) spleen; D: (Lv1) right liver, (Lv2) left liver

The malformed dead female puppy had a single head (monocephalus). The cranium was normal with no fissure in the medial line detected. One mandible, a tongue and a single row for the molar pads were present. The palate was normal with no cleft evident (Fig. 1A). Necropsy examination confirmed that the twins were joined at the lumbosacral region (rachipagus) where the bifurcation (dipygus) commenced (Figs. 2A and 2B). There were two separate pelvises (Fig. 1).

The dead puppy had three upper limbs, a right and left, and a third limb in the dorsal region where the bifurcation began (Fig. 1C). This extra limb was attached to the caudal region of the lumbar vertebrae and was surrounded by a mass of muscular tissue. Each upper limb of this puppy was completely developed without any apparent anomalies. The subsequent caudal duplication appeared symmetrical (four lower limbs and two tails), (Figs. 1B, 1C and 1D).

The thoracic and abdominal cavities were divided by a single diaphragm (Fig. 2A). There was a single sternum, a fully developed heart and two collapsed lungs (Fig. 2B). The anterior abdominal cavity contained a single stomach and spleen. (Fig. 2C). Distal to the stomach, the small intestine had normal morphological characteristics in the region of duodenum and jejunum, but in the distal portion of the ileum a bifurcation flowed into a duplication of the caecum, colon and rectum.

**Fig. 3:**
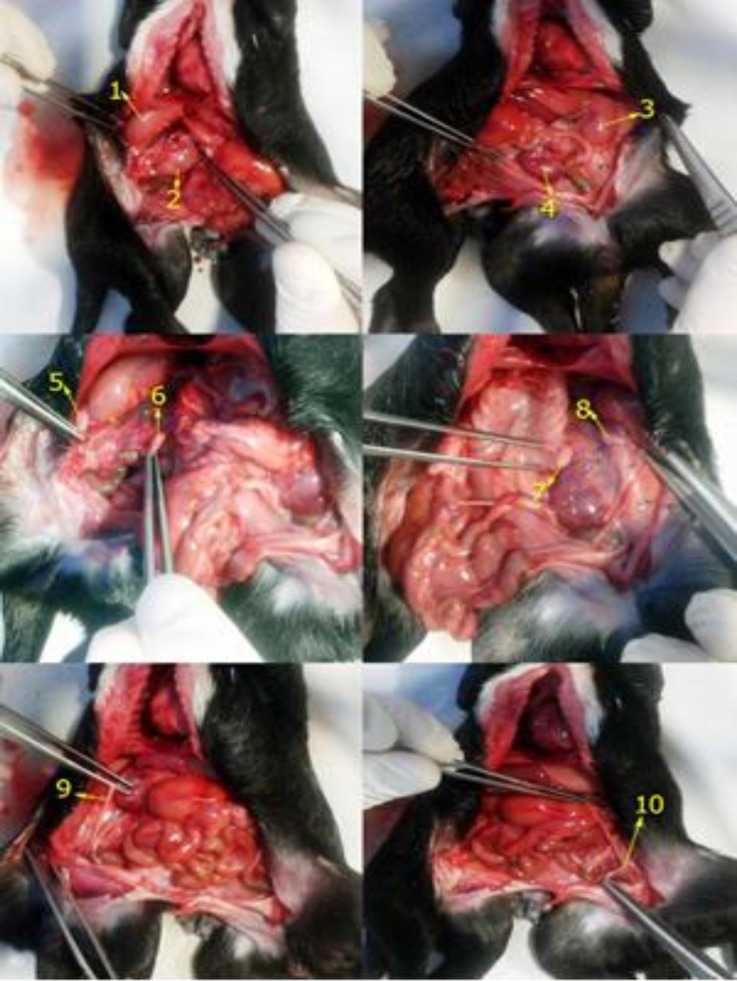
Organs of genitourinary system. Organs of right bifurcation: (1) right kidney, (2) left kidney, (5) right ovary, (6) left ovary, (9) uterus; Organs of left bifurcation; (3) left kidney, (4) right kidney, (7) right ovary, (8) left ovary, (10) uterus

There were two complete urinary systems (four kidneys, and two bladders) and two complete genital systems (four ovaries and two uteri) were noted (Fig. 3). The liver also was found to be duplicated, each organ fully lobed and with separate gallbladders (Fig. 3).

Considering the morphophysiological malformation described above, this puppy would be classified as Monocephalus rachipagus tribrachius tetrapus.

## Discussion

Abnormalities of the structure or function of tissues or organs which are present at birth are termed congenital defects. A malformation is a type of congenital defect which is intrinsic to the embryological differentiation or development of the structure.^4^ Conjoined twinning is one of the most interesting malformations and has been reported in humans,^6^^,^^17^ birds, ^7^^,^^8^ sheep,^9^ cat,^13^ cattle,^18^^-^^20^ and goat.^10^^,^^20^ In humans, the incidence has been reported to be between one in every 50,000 to 100,000 live births.^6^ Congenital duplications occur more frequently in cattle than other domestic species.^18^^,^^21^ Nevertheless in dog populations developmental defects are reported to affect approximately 6.0% of pups and are reported infrequently in cats.^4^

The malformation described in this report is termed monocephalus dipygus, considered as a form of duplication or doubling anomaly whose etiology is not clear. Dipygus is also known as ‘teras catadidymum’, ‘monster twinned below’ or ‘quadripus dibrachius’. This malformation is generally considered to be due to an error in blastogenesis arising from incomplete fission of a single zygote.^1^^,^^5^

Similar rare duplications originating in caudal region have been reported in dogs: monocephalus rachipagus dibrachiustripus,^14^ monocephalusthoracopagus tetrabrachius tetrapus,^15^ monocephalus thoracopagus tetrabrachius tetrapus and monocephalus thoracopagus dibrachius tetrapus reported by Oviedo et al.^16^ Whilst the supernumerary tribrachius limb also was reported in sheep by Mazaheri et al.,^9^ no reports of any tribrachius in the dog were found in the literature.

Twins joined at the lumbosacral region are named rachipagus. Rachipagus twins are those, which arise dorsally near the vertebral canal or presumably near the vertebral arches and are considered to be a rare type of conjoined twinning.^22^ Some reports of rachipagus have been reported in humans.^22^^,^^23^

Genetic, environmental and drugs may play a role in triggering the condition.^4^^,^^12^^,^^14^ One example of the genetic origin of malformation was described in a report by Freick et al.,^20^ where evidence was found for a monozygotic origin of incomplete caudal duplication in a German Holstein calf.

A number of drugs have been classified according to their teratogenic risk. The most widely used classification system is that issued by FDA where drugs are classed into five groups (A, B, C, D, X).^24^

The dam was a rescued stray dog so her ancestry is unknown however her dominant phenotype was that of the German shepherd breed. There was no history of the dam having been exposed to known teratogenic substances during the pregnancy. Until the 1960s most congenital defects were considered genetically related, but now environmental factors are recognized as the major causes of malformations.^10^ This pregnancy did occur during the hottest part of the year so ambient temperature may have played a role in the causation of this malformation.

According to Dominguez et al.,the presence of malformations is considered to be one of the causes of dystocias.^25^ In this case, the oversize of the malformed fetus in the birth canal caused an obstructive dystocia. In humans,^6^ goat,^10^ and sheep,^9^ similar presentations required a caesarian delivery but in the cat,^13^ the unassisted queen delivered the dipygus kitten vaginally.

To the best of the authors’ knowledge, this is the first report in the literature of Monocephalus rachipagus tribrachius tetrapus in a dog. It would be ideal to perform radiographic studies pre-autopsy when investigating similar cases in the future so as to allow for a greater appreciation and documentation of the individual anatomic variations present in each malformed individual.

## References

[1] Spencer R (2001). Parasitic conjoined twins: External, internal (fetuses in fetu and teratomas), and detached (acardiacs). Clin Anat.

[2] Lanteri G, Macri F, Marino F (2013). A rare case of deradelphus cephalo-thoracoomphalopagus in lamb. Anat Histol Embryol.

[3] Schneevoigt J, Bahramsoltani M, Gerlach K (2014). Parapagus conjoined twin calf: A case study - focused on CT and cardiac abnormalities. Anat Histol Embryol.

[4] McGeady TA, Quinn PJ, FitzPatrick ES (2006). Teratogens. Veterinary embryology.

[5] Sadler TW (2011). Langman’s medical embryology.

[6] Prieto MC, Brea AF, López CE (2004). Dipygus: A case report [Spanish]. Rev Obstet Ginecol Venez.

[7] Corbera JA, Morales I, Arencibia A (2012). Caudal duplication (dipygus) in a Rock Pigeon (columba livia). Eur J Anat.

[8] Hirschberg RM, Saleh M, Kaiser S (2012). Polymelous layer chick displaying additional malformations of the hind gut: Case report and in-depth review of related literature. Anat Histol Embryol.

[9] Mazaheri Y, Nourinezhad J, Ranjbar R (2014). A case of conjoined twins (thoraco-omphalopygopagustribrachius tetrapus) in lamb. Vet Res Forum.

[10] Buhari S, Yakubu AS, Jibril A (2008). Monocephalus, thoracopagus and dipygus twins in Sokoto Red goat. Sokoto J Vet Sci.

[11] Corbera JA, Arencibia A, Morales I (2005). Congenital duplication of the caudal region (monocephalus dipygus) in a kid goat. Anat Histol Embryol.

[12] Shojaei B, Mohebbi E, Hashemnia S (2012). Caudal duplication (monocephalus tripus dibrachius) in a kid goat. Eur J Anat.

[13] Seavers AM (2009). Monocephalusdipygusparapagus: A suspected case of complete caudal duplication in a British blue kitten. J Feline Med Surg.

[14] Mazzullo G, Monteverde V, Macri F (2007). Incomplete caudal duplication in a puppy: Gross and radiological observations. J Small Anim Pract.

[15] Nottidge HO, Omobowale TO, Olopade JO (2007). A case of craniothoracopagus (monocephalus thoracopagus tetrabrachius) in a dog. Anat Histol Embryol.

[16] Oviedo TS, González MT, Oviedo MP (2008). Monocephalus dipygus in puppies. Report of two cases [Spanish]. Rev MVZ Cordoba.

[17] Al Alayet YF, Samujh R, Lyngdoh TS (2014). An extremely rare case of classic complete caudal duplication: Dipygus. J Indian Assoc Pediatr Surg.

[18] Masoudifard M, Shojaei B, Hashemnia S (2008). Monocephalus dipygus (tetrapus dibrachius) in a Calf. OIE.

[19] Abt DA, Croshaw JJ, Hare WC (1962). Monocephalus dipygus parasiticus and other anomalies in a calf. J Am Vet Med Assoc.

[20] Freick M, Behn H, Hardt M (2014). Monozygotic incomplete caudal duplication in a German Holstein calf. Vet Rec Case Rep.

[21] Roberts SJ (1986). Veterinary obstetrics and genital diseases (Theriogenology).

[22] Kota RR, Srirampur S, Kannaiyan L (2012). Parasitic twinning - Varied presentations. J Dr NTR Univ Health Sci.

[23] Sanoussi S, Rachid S, Sani CM (2010). Rachipagus: A report of two cases - thoracic and lumbar. J Surg Tech Case Report.

[24] Pérez-Landeiro A, Allende-Bandrés MA, Agustín Fernández MJ (2002). Teratogenesis: Clasificaciones. Farm Hosp.

[25] Dominguez JC, Pena FJ, Castro B (1994). Parturition and dystocia in dog and cat [Spanish]. Clin Vet Peq Anim.

